# Quality of prescription writing in Brazilian primary health care

**DOI:** 10.1017/S1463423623000415

**Published:** 2023-07-31

**Authors:** Almária Mariz Batista, Zenewton André da Silva Gama, Pedro Jesús Saturno Hernández, Dyego Souza

**Affiliations:** 1 Multicampi School of Medical Sciences, Federal University of Rio Grande do Norte, Caicó, Brazil; 2 Instituto Nacional de Salud Pública, Cuernavaca, México; 3 Department of Collective Health, Federal University of Rio Grande do Norte, Natal, Brazil; 4 Graduate Programme in Health Sciences, Federal University of Rio Grande do Norte, Natal, Brazil

**Keywords:** drug prescriptions, medical writing, patient safety, primary health care, quality of health care

## Abstract

**Objective:**

To evaluate the quality of prescription writing in the context of public primary health care.

**Background:**

Prescription errors are one of the leading patient safety problems in primary care and can be caused by errors in therapeutic decisions or in the quality of prescription writing.

**Methods:**

Cross-sectional observational study conducted in a municipality in Northeastern Brazil. The assessment instrument (including 13 indicators and one composite indicator) was applied to a representative sample of drug prescriptions from the 24 Family Health Teams providing Primary Health Care in the municipality, dispensed in January 2021. Estimates of compliance and their 95% confidence intervals and graphical analysis of frequencies are assessed globally and stratified by dispensing units and prescribers.

**Findings:**

The average composite prescription writing quality on a 0-100 scale was 60.2 (95% CI 57.8–62.6). No quality criteria had 100% compliance. The highest compliance rates were found for ‘frequency of administration’ (98.9%) and ‘identification of the prescriber’ (98.9%). On the other hand, ‘recorded information on allergy’ (0.0%), ‘patient’s date of birth’ (1.7%), ‘nonpharmacological recommendations’ (1.7%), and ‘guidance on the use of the drug’ (25%) were the indicators with lower compliance, contributing to 69% of the noncompliances found. The type and frequency of the errors in the quality of prescription writing uncovered in this study confirm the continuing need to tackle this problem to improve patient safety. The results identify priority aspects for interventions and further studies on the quality of prescription writing in the context of Primary Health Care in Brazil.

## Introduction

Medication errors are a significant cause of morbidity and mortality worldwide, a serious public health problem (Jha *et al.*, 2013; Organization for Economic Cooperation and Development (OECD), 2017). Medication errors are considered the leading patient safety incident in primary care in which 30%–50% of unnecessary healthcare-related harm occurs (Organization for Economic Cooperation and Development (OECD), 2018). In the early nineties, the World Health Organization (WHO) published the Guide to Good Prescribing (World Health Organization, 1994) and more recently called for complete attention to this problem with the Global Patient Safety Challenge on Medication Safety (World Health Organization, 2017).

The medication process is complex because it involves different professionals and multiple steps, which include selection, prescription, dispensing, administration, and monitoring of the use of drugs (Nadzan, 1998). Additionally, medication practices are not always guided by technical issues, as they involve beliefs and social and cultural pressures that can lead to medication errors.

Studies show that prescription is most susceptible to primary care medication errors (Bates *et al.*, 1995; Montserrat-Capella *et al.*, 2015; Olaniyan *et al.*, 2015). These errors may be due to weaknesses in the processes of therapeutic decision and prescription writing (Brasil, 2013; Dean *et al.*, 2000). Prescription writing encompasses structuring this document following the prescriber information, the patient information, and the technical specifications of prescribed drugs, aligned with internationally standardized abbreviations/acronyms/symbols (World Health Organization, 1994).

Despite the existence of national and international recommendations on the quality of prescription writing (World Health Organization, 1993, 1994, 2018; Barber, 1995; Cohen, 1999; Andersen, 2006; Brazil, 2013), as well as studies based on these recommendations (Meyer, 2000; Imran *et al.*, 2020; Dyasanoor & Urooge, 2016; Joshi *et al.*, 2016; Mohammed Al-Worafi *et al.*, 2018; Varghese *et al.*, 2018; Silva Júnior & Batista, 2019; Weldemariam *et al.*, 2020; Silva *et al.*, 2021; Jota & Batista, 2022), there is still a lack of knowledge about studies on the quality of prescription writing based on validated indicators in the context of primary care.

This scarcity of studies is more important in the context of primary care, particularly in Brazil, where funding is often insufficient to provide, for example, electronic prescriptions. This information technology contributes to improving the quality of prescription writing (Joshi *et al.*, 2016). However, it is necessary to consider that this resource is not free from prescription errors (Odukoya *et al.*, 2014; Joshi *et al.*, 2016; Yousuf *et al.*, 2016; Brown *et al.*, 2017; Schiff *et al.*, 2018; Nurfitria *et al.*, 2019; Abramson, 2015).

One possibility to evaluate the quality of prescription writing in primary care is the application of criteria that cover prescriber, patient, and drug information (World Health Organization, 1993, 1994, 2018; Barber, 1995; Cohen, 1999; Andersen, 2006; Brasil, 2013). This is intended to identify strengths and weaknesses of drug prescription in primary care, compare services, and evaluate the performance of drug prescribers and dispensers concerning their responsibilities regarding prescription quality.

In this context, the objective of this study was to evaluate the quality of prescription writing in the context of primary care. The results can contribute to understanding this problem and identify targets and studies to improve this relevant issue for patient safety.

## Method

### Design

Observational and cross-sectional study with prescriptions conducted in a municipality in Northeastern Brazil in January 2021, using an instrument developed and validated to evaluate the quality of prescription writing.

### Context

This study is part of a research and extension project to improve drug prescription quality in primary health care, based on the cooperation between a Brazilian federal university and the health department of a municipality in Northeastern Brazil, Caicó-RN, whose population is estimated at 68 726 inhabitants and the human development index (HDI) is 0.710 (Instituto Brasileiro de Geografia e Estatística, 2021).

It takes place within the scope of primary health care, which is offered with public services from the Brazilian Sistema Único de Saúde (SUS). The SUS is made up of a set of health actions and services provided by federal, state, and municipal agencies and public institutions, belonging to the direct and indirect administration and foundations maintained by the public power, based on the principles of universality, equity, and integrality (Brasil, 1990).

From this perspective, primary care has family health as its priority strategy for expanding and consolidating following the SUS precepts. Family Health Teams (FHT) comprise at least a physician, preferably from the family and community medicine specialty; a nurse; a nursing assistant; a technician; and a community health worker (Brasil, 2017).

The Caicó-RN municipality has 24 FHT distributed in 18 primary health care units (PHCU). Concerning drug dispensing, it has six pharmacies, which will henceforth be called drug dispensing/distribution units (DUs). The patient is free to pick up the medication in any DU, regardless of the FHT/PHCU where he received healthcare, except for drugs that act on the central nervous system level, whose dispensation/distribution is centralized in DU 1, 4, and 6.

### Participants

The study units were drug prescriptions prepared by 24 physicians working in the 24 FHTs of the municipality under study in January/2021. The unit of analysis is prescription. Thus, for example, for the ‘pharmaceutical form’ indicator, regardless of the number of prescribed drugs in each prescription, if the specification of one single drug is not in compliance with the ‘pharmaceutical form’ indicator, the prescription will be considered noncompliant for that indicator.

Prescriptions prepared by physicians not located in these PHCU and nonmedical professionals and prescriptions whose evaluation was not feasible due to readability issues were excluded. For this study, readability was not considered an indicator because, in the case of readability problems, it is unfeasible to apply the other indicators due to the impossibility of reading the prescription content. In addition, the readability test requires face-to-face administration of a questionnaire to at least 20 users, preferably from the target population of the drug, and excludes health professionals, in order to avoid results biased by their specialized knowledge (European Medicine Agency, 2009; The Heads of Medicines Agencies, 2011). On the other hand, considering legibility as an exclusion criterion also adds the possibility of applying it as a prerequisite for assessing the quality of prescription writing.

The sampling to select the prescriptions was random, stratified by DUs, and nonproportional. Regardless of the prescription volume, in order to ensure comparability among these DUs, the target sample was 30 prescriptions randomly selected in each of the six DUs, a minimum standard for frequency calculation and comparison among groups (Saturno-Hernández, 2015), totaling 180.

### Variables

We assess 13 indicators (with their respective specific scores) and one composite indicator (total score). These indicators were considered adequate by an expert committee. They were previously piloted to assess their validity, reliability, and usefulness in evaluating the quality of prescription writing in the context of primary care (Batista *et al.*, 2022).

For each of these indicators, there is an operational definition. For the application of the ‘active principle’, ‘concentration’, ‘dose’, and ‘route of administration’ indicators, international recommendations on abbreviations/acronyms/symbols were considered (Brasil, 2013; The Joint Commission, 2021) (Table 1).

**Table 1.** Definitions and clarifications about validated indicators

Simple indicator	Definition/Clarifications	Score
Patient’s date of birth	Day/month/year.	
Prescriber’s identification	It covers professional registration number at the Regional Council, profession, name, and surname of the prescriber. The intermediate words to the first and last names can be constituted by the initial letters.	6.4
Allergy report record	Record of information about the fact that the user is allergic to certain medication(s). It must be included in the prescription, regardless of the drug involved being present in the prescription.	4.2
Medicine included in the institutional list officially approved	Component of the standard list of drugs of an institution, standardized, preferably, according to the epidemiological profile and the best scientific evidence of efficacy, safety, and cost-effectiveness. As an alternative to the standard list of a specific institution, the standard list of municipal, state, or national essential drugs can be used as a basis, considering the most appropriate to the context.	7.0
Active principle	It is the Common Brazilian Denomination (DCB, as per its Portuguese acronym) or, in its absence, the Common International Denomination (CID). Do not use abbreviated drug names or chemical formulas (e.g., MgSO_4_). Write the nomenclature in full (Brasil, 2013; The Joint Commission, 2021).	4.8
Concentration	It corresponds to the amount of active ingredient contained in each dosage unit. For solid pharmaceutical forms, one dosage unit corresponds to one unit of the pharmaceutical form (e.g., 1 tablet). For semi-solid and liquid dosage forms, one dosage unit corresponds to one measurement unit (e.g., 1 ml, 1 g). Do not use the acronyms U, u, and UI, as they can be confused with zero or four or cc or IV or 10, write international units. Do not use acronyms mcg or µg, write microgram. Do not use zero on the right or omit zero on the left, as the decimal place can go unnoticed; write X mg and 0.X mg, respectively. For doses or volumes with fractional numbers (e.g., 2.5 mL), check on both sides of the prescription that the comma is well positioned and clear. Do not use a period in place of a comma (Brasil, 2013; The Joint Commission, 2021).	5.8
Dose	Quantity of medication to be administered at each time of use. Consisting of a numerical value and a measurement unit. As a measurement unit, consider the name of the solid pharmaceutical form or drops or abbreviations/acronyms/symbols recommended nationally and/or internationally. Do not use the acronyms U, u, and UI, as they can be confused with zero or four or cc or IV or 10, write international units. Do not use acronyms mcg or µg, write microgram. Do not use zero on the right or omit zero on the left, as the decimal place can go unnoticed; write X mg and 0.X mg, respectively. For doses or volumes with fractional numbers (e.g., 2.5 mL), check on both sides of the prescription that the comma is well positioned and clear. Do not use a period in place of a comma (Brasil, 2013; The Joint Commission, 2021).	9.2
Pharmaceutical form	Final physical form of the drug after mixing between active ingredients and excipients during the production process.	9.9
Route of administration	Gateway through which the medication is administered in order to reach its place of action (e.g., oral, intramuscular, intravenous, etc.). Prefer using EV (intravenous) instead of IV (intravenous), due to the risk of misinterpretation of IV as IM (Brasil, 2013; The Joint Commission, 2021).	8.9
Frequency of administration	Number of times, considering the 24-hour period in which each dose of medication must be administered (e.g., every 12 h, etc.).	9.2
Duration of treatment	Length of time the drug should be used (e.g., for 10 days, continuous use, etc.). If the prescription has more than one drug, consider it in compliance for this item only if all prescription drugs meet the indicator.	9.9
Directions on the use of the drug	Best period for drug administration (e.g., morning/evening, before/after meals, fasting, after bathing), duration of drug administration (e.g., minutes until container content is finished), thin layer application (e.g., topical use), drug dilution, and instruction for the use of an inhaled device, among others necessary for the user’s understanding of how to use the drug.	9.7
Nonpharmacological recommendations	Information concerning nondrug treatment (e.g., diet, physical activity, etc.).	9.9
Composite indicator	Clarifications for the measurement	
Prescription quality	(1) To evaluate a prescription, the calculation is the sum of the scores of each of the 13 simple indicators of the QualiPresc instrument in case of compliance. Quality QualiPresc = ∑quality scores I1-I13 (2) To evaluate aggregated data, it is the average of the sum found in each prescription. QualiPresc quality = ∑quality scores I1-I13 × total prescriptions/time	5.3

In addition to the quality of prescription writing indicators, prescribing physicians, DUs, pharmacological classes of prescribed drugs, and the number of drugs/prescriptions were noted for the analysis.

### Data collection

Drug prescriptions stored in all six DUs in that city constituted units of analysis.

A previously trained pharmacist evaluated these documents using the QualiPresc validated instrument (Batista *et al.*, 2022) from February to March 2021. The process of selection/verification of compliance of the selected prescriptions with the 13 QualiPresc indicators covered an average time of 3h17min per DU.

The evaluation of the compliance of the prescriptions with the indicator ‘medicine included in the institutional list officially approved’ was based on the most current version of the National List of Essential Medicines (Brasil, 2020).

The pharmacological classification of prescribed drugs was based on the Anatomical Therapeutic Classification (ATC) system (World Health Organization, 2013).

### Data analysis

For the 13 indicators, the relative frequencies of compliance and their 95% confidence intervals (95% CI) were estimated for the entire municipality and the six DUs. In the case of the general estimate, the estimate and variance calculations were adjusted following the appropriate formulas for nonproportional sampling (Saturno-Hernández, 2015). In addition, there was an analysis of priorities for intervention using a Pareto chart of noncompliances, identifying the indicators that contributed most to noncompliances, and the cumulative frequency concerning the total noncompliances of evaluated indicators in the sample. For the composite indicator, the average of the total scores of each evaluated prescription was calculated, considering a 95% CI.

Prescription writing quality level and average prescription writing quality level (concerning the total prescriptions evaluated, by DU and by prescriber, along with identifying priority quality defects for intervention) constitute the outcomes of this study.

The six DUs were compared through bivariate analysis with the chi-square test or the likelihood ratio. The significance level adopted was 5%. Additionally, a graphical analysis of the frequency distribution of prescription quality scores was performed using histograms and box plots.

For the quality composite indicator, after verifying that the data from each DU did not present a normal distribution based on the Kolgomorov–Smirnov test, the nonparametric Kruskal–Wallis test was applied to compare medians.

For the statistical analysis, we used the SPSS software, version 25.0.

### Patient and public involvement

No patient was involved.

### Ethical aspects

This project was approved by the Research Ethics Committee of the Onofre Lopes University Hospital (CEP/HUOL) under number 64367517.3.0000.5292.

## Results

### General analysis of drug prescriptions

We selected 329 prescriptions, of which 149 did not meet the inclusion criteria. The reasons for exclusion were prescriptions by nonmedical or nonprimary care medical professionals (130 prescriptions) and legibility problems that made it impossible to assess the indicators (19 prescriptions). Our final sample to assess was 180 prescriptions.

The average number of drugs by prescription was 1.8. There was a predominance of pharmacological classes of antimicrobials for systemic use (20.2%), antihypertensive (18.5%), and analgesic/anti-inflammatory (13.1%).

**Figure 1.**
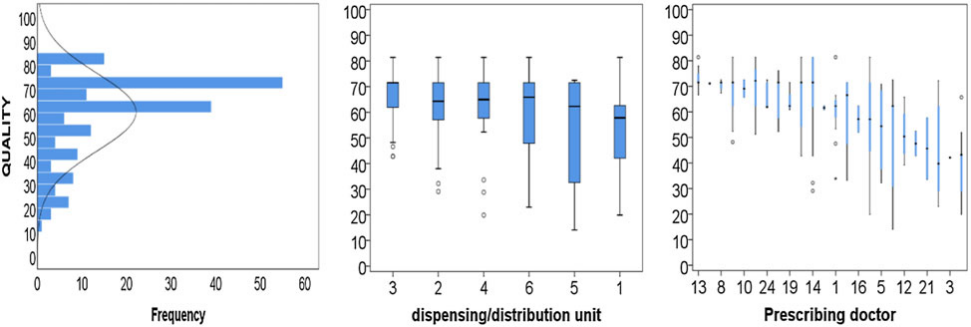
Matrix-plot of comparative analysis of the distribution of the quality level of the evaluated prescriptions, January/2021 (*n* = 180).

The average level of prescription quality on a 0–100 scale was 60.2 (95% CI 57.8–62.6). The analysis of the frequency distribution of the quality levels of the evaluated prescriptions reveals an asymmetric distribution to the right, where there is a predominance of more adequate prescriptions. This demonstrates that most of these prescriptions have a quality level above the average. On the other hand, all analyses indicate a substantial variability within a situation of quality that can be improved (Figure 1).

As for prescription compliance, a higher prevalence of compliance was found for the indicators ‘frequency of administration’ (98.7%) and ‘prescriber’s identification’ (98.3%) (Table 2).

**Table 2.** General and stratified analysis of compliance in the dispensing/distribution units of Caicó, January/2021

Indicator	% Adjusted Compliance (95% CI) N = 3384 *n* = 180	% Compliance (95% CI)						*P*
		DU1 N = 659 *n* = 30	DU2 N = 407 *n* = 30	DU3 N = 302 *n* = 30	DU4 N = 683 *n* = 30	DU5 N = 223 *n* = 30	DU6 N = 1110 *n* = 30	
Patient’s date of birth	2.9 (0.0–6.1)	0.0 (0.0–0.0)	0.0 (0.0–0.0)	0.0 (0.0–0.0)	3.3 (0.0–9.8)	0.0 (0.0–0.0)	6.7 (0.0–15.6)	0.212
Prescriber’s identification	98.3 (95.8–100.0)	96.7 (90.2–100)	100 (100.0–100.0)	100 (100.0–100.0)	100 (100.0–100.0)	100 (100.0–100.0)	96.7 (90.2–100)	0.488
Allergy report record	0.0 (0.0–0.0)	0.0 (0.0–0.0)	0.0 (0.0–0.0)	0.0 (0.0–0.0)	0.0 (0.0–0.0)	0.0 (0.0–0.0)	0.0 (0.0–0.0)	–
Medicine included in the institutional list officially approved	72.2 (64.6–79.8)	63.3 (46.1–80.6)	70 (53.6–86.4)	96.7 (90.2–100.0)	73.3 (57.5–89.2)	76.7 (61.5–9.18)	70 (53.6–86.4)	0.060
Active principle	74.8 (67.3–82.3)	73.3 (57.5–89.2)	86.7 (74.5–98.8)	93.3 (84.4–102.3)	76.7 (61.5–91.8)	83.3 (70–96.7)	63.3 (46.1–80.6)	0.065
Concentration	81.1 (74.6–87.6)	70 (53.6–86.4)	86.7 (74.5–98.8)	90 (79.3–100.0)	83.3 (70–96.7)	73.3 (57.5–89.2)	83.3 (70–96.7)	0.311
Dose	79.7 (73.1–86.3)	66.7 (49.8–83.5)	86.7 (74.5–98.8)	90 (79.3–100.0)	80 (65.7–94.3)	73.3 (57.5–89.2)	83.3 (70–96.7)	0.215
Pharmaceutical form	85.1 (79.1–91.1)	76.7 (61.5–9.18)	93.3 (84.4–100.0)	96.7 (90.2–100.0)	90 (79.3–100.0)	73.3 (57.5–89.2)	83.3 (70–96.7)	0.046
Route of administration	58.4 (50.6–66.2)	36.7 (19.4–53.9)	96.7 (90.2–100.0)	96.7 (90.2–100.0)	66.7 (49.8–83.5)	16.7 (3.3–30)	50 (32.1–67.9)	<0.001
Frequency of administration	98.7 (96.9–100.0)	93.3 (84.4-100.0)	100 (100.0–100.0)	100 (100.0–100.0)	100 (100.0–100.0)	100 (100.0–100.0)	100 (100.0–100.0)	0.201
Duration of treatment	72.2 (65.0–79.4)	70 (53.6–86.4)	46.7 (28.8–64.5)	76.7 (61.5–9.18)	80 (65.7–94.3)	73.3 (57.5–89.2)	76.7 (61.5–9.18)	0.057
Directions on the use of the drug	26.7 (19.2–34.2)	23.3 (8.2–38.5)	26.7 (10.8–42.5)	20 (5.7–34.3)	23.3 (8.2–38.5)	23.3 (8.2–38.5)	33.3 (16.5–50.2)	0.890
Nonpharmacological recommendations	1.3 (0.0–3.1)	3.3 (0.0–9.8)	0.0 (0.0–0.0)	0.0 (0.0–0.0)	3.3 (0.0–9.8)	0.0 (0.0–0.0)	0.0 (0.0-0.0)	0.217
**Composite indicator**	Average of compliance (95% CI)		Average (95% CI)					
Quality	60.2 (57.8–62.6)	53.0 (46.7–59.3)	63.4 (58.0–68.7)	68.2 (64.0-72.4)	62.4 (57.1–67.6)	54.0 (46.8–61.3)	60.0 (54.0-66.0)	0.011

CI: confidence interval; DU: drug dispensing/distribution unit; N: universe size; *n*: sample size.

**Figure 2.**
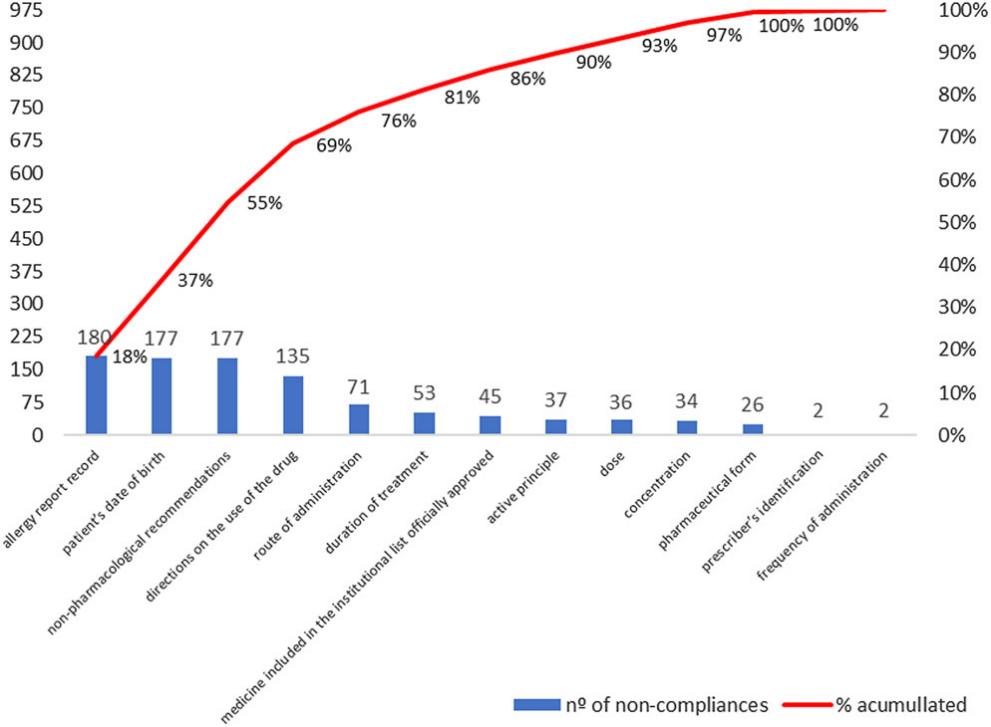
Frequencies of noncompliance of QualiPresc indicators, January/2021 (*n* = 180).

**Figure 3.**
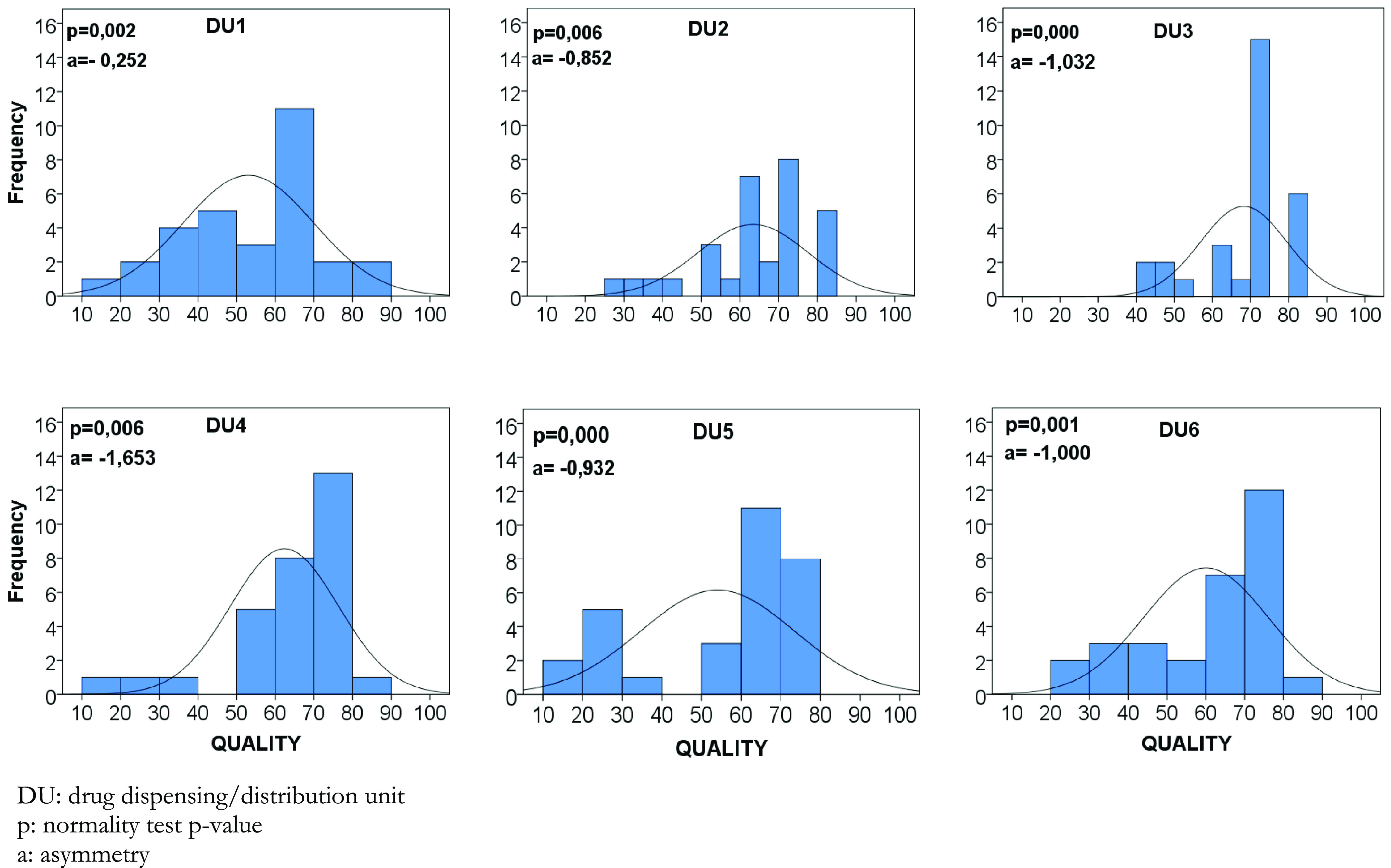
Matrix-plot of distribution of drug prescription quality levels by dispensing/distribution units, January/2021 (*n* = 30).

On the other hand, ‘allergy report record’ (0.0%), ‘nonpharmacological recommendations’ (1.3%), ‘patient’s date of birth’ (2.9%), and ‘directions on the use of the drug’ (26.7%), the latter the highest score (9.9) among the QualiPresc constituents, were the indicators with the lowest prevalence of compliance. This contributes to 69% of the nonconformities found in the evaluated prescriptions, from 30.8% of the indicators that make up QualiPresc (Figure 2–3, Table 2).

### Stratified analysis of drug prescriptions

The analysis of DU and prescribers also demonstrates variability in the quality levels of drug prescriptions, where quality levels between 10 and 85 are demonstrated, with the lowest levels being related to DU1 53.0 (46.7–59.3) and DU5 54.0 (46.8–61.3) and prescribers 4, 11, 14, 20, and 22 (Figure 2). In this sense, the Kruskal–Wallis test found a statistically significant difference among the average levels of drug prescription quality (*P* = 0.011).

Additionally, the frequency distribution of DU’s quality levels of the evaluated prescriptions demonstrates that the histograms are asymmetric to the right. However, no prescription reached the highest quality level (Figure 3).

Furthermore, considering the stratified analysis of compliance of prescriptions by indicator, it appears that this statistically significant difference is related to the ‘pharmaceutical form’ (*P* = 0,046) and ‘route of administration’ (*P* < 0·001) indicators (Table 2).

## Discussion

This study evaluates the quality of prescription writing in the context of primary care using a validated instrument (Batista *et al.*, 2022). In this way, it contributes to benchmarking and identifying opportunities for improvement in this relevant topic of patient safety in primary care. Furthermore, the weaknesses identified in this study show that there is a long path to adherence to good prescription practices regulated for this context.

The study identified an average quality of prescription writing compliance of 60% (score 60 out of 100), mainly due to the four most prevalent noncompliant indicators: ‘allergy report record’ (0.0%), ‘nonpharmacological recommendations’ (1.3%), ‘patient’s date of birth’ (2.9%), and ‘directions on the use of the drug’ (26.7%). These were responsible for 69% of the noncompliances found in the evaluated prescriptions. Drug prescribing professionals tend to disregard prescription writing criteria to the detriment of therapeutic decision criteria (Andersen, 2006). However, flaws in writing prescriptions also tend to compromise the safety and effectiveness of drug therapy (World Health Organization, 1994).

The problems identified gain relevance when considering the pharmacological classes of the prescription drugs identified in this study. The most prevalent pharmacological class was antimicrobials for systemic use, a priority in the WHO Global Challenge Medication without Harm (World Health Organization, 2017). Furthermore, 12.8% of the evaluated prescriptions contained potentially dangerous drugs (Instituto para Práticas Seguras No Uso de Medicamentos, 2015), also a priority in the WHO Global Challenge (World Health Organization, 2017), with a predominance of oral drugs used in diabetes. In this case, the patient or caregiver must be informed, in print and verbal form, of the therapeutic scheme and prescribed procedures (Cohen *et al.*, 2006; Ministerio de Sanidad y Consumo *et al.*, 2007).

### Opportunities for improvement in the quality of prescription writing

The indicators with the lowest compliance were ‘allergy report record’ (0.0%), ‘nonpharmacological recommendations’ (1.3%), ‘patient’s date of birth’ (2.9%), and ‘directions on the use of the drug’ (26.7%). They contradict recommendations such as step four of the WHO Six-Step Method for Rational Prescribing (writing a prescription correctly) (World Health Organization, 1994) and the WHO Curriculum Guide for Patient Safety (World Health Organization, 2011), which advocates adequate communication in the prescription writing process.

None of the evaluated prescriptions had a record of drug allergy. Nonetheless, antimicrobials and analgesics/anti-inflammatories, the most prevalent pharmacological classes in the evaluated prescriptions, stand out as potential causes of allergic reactions.

The prescription’s omission of ‘nonpharmacological recommendations’ can hinder achieving essential therapeutic goals (Brasil, 2013). Notably for chronic health problems such as systemic arterial hypertension, for which, in this case, antihypertensive drugs also stand out as the prevalent pharmacological class. The failure regarding this indicator can be explained by the culture of medicalization in health care, which tends to overvalue the use of medications to the detriment of nonpharmacological therapies.

The indicator ‘directions on the drug use’ also has serious repercussions for the patient. This is additional information such as the best period for drug administration (e.g., morning/evening, before/after meals, fasting, after bathing), duration of drug administration (e.g., minutes until container content is finished), thin layer application (e.g., topical use), drug dilution, and instruction for the use of an inhaled device, among others necessary for the user’s understanding of how to use the drug. These additional directions are even more critical in primary care because, unlike tertiary care, drug administration is predominantly performed by the user. Furthermore, this indicator scores higher than the other indicators. This makes it a priority to target interventions to improve drug prescription quality, even among the four with the lowest compliance.

In this context, implementing electronic prescriptions can be an opportunity for improvement. In this case, it is necessary to consider that this can reduce prescription errors as long as its implementation is adequately planned and monitored and its access is authorized through prior training by specialists in patient safety (Prgomet *et al.*, 2017; Rosa *et al.*, 2019). Additionally, an evaluation of its effects in primary care found a reduction in prescribing errors if applied to a limited number of potentially dangerous drugs and through physician-pharmacist communication (Lainer *et al.*, 2013).

### Indicators with high compliance

From a positive perspective, the indicators with the highest compliance were ‘prescriber’s identification’ (98.3%) and ‘frequency of administration’ (98.7%).

On the other hand, the indicator ‘medicine included in the institutional list officially approved’, showing compliance of 72.2%, seems to be underreported. Health needs assessment and prescribing behavior were items influenced by pharmaceutical marketing (Vargas-Pelaez *et al.*, 2019). Suppose the prescribed drug is unavailable in DUs due to shortages or noncompliance with the list of essential drugs. In that case, the user does not carry out the pharmacological treatment or conduct the prescription for purchase via a government program, own resources, or judicialization (Biehl *et al.*, 2012; Catanheide *et al.*, 2016; Vargas-Pelaez *et al.*, 2019; Oliveira *et al.*, 2021). This way, these prescriptions remain in the patient’s possession instead of being filed in DUs. This may have resulted in underestimating the noncompliance of the ‘medicine included in the institutional list officially approved’ indicator. This suggests a compromise of the drug selection step.

### Stratified analysis of drug prescriptions

The evaluation of drug prescriptions also considered their comparison between drug DUs. In this sense, a statistically significant difference among DUs regarding the level of prescription quality presupposes noncompliance with Good Pharmaceutical Practices was found. These include evaluating the prescription by the pharmacist through therapeutic aspects and contacting prescribers about individual prescriptions (Organização Pan-Americana de Saúde/Organização Mundial de Saúde/Conselho Federal de Farmácia OPAS/OMS/CFF, 1994).

Dispensing has been neglected, nationally and internationally, as simple drug delivery needed a reorientation in which the pharmaceutical clinic is a socio-technical activity of dispensing (Angonesi, 2008; Leite *et al.*, 2017), which will best qualify this practice for evaluating aspects of the therapeutic decision and prescription writing. As for DUs, one, four, and six filed a higher number of prescriptions, as they also centralize the drugs dispensing/distribution that act on the central nervous system. This increase in demand can overwhelm the pharmacist, compromising the assessment of prescription quality via dispensing.

### The internal and external validity of the results

This study has internal validity because the methodological precautions of a random sample selection and adjustment of prescription quality estimates provide generalizability for the entire studied municipality. However, caution should be exercised when extrapolating these results to other Brazilian municipalities or other countries because contextual variations in primary care can influence the study’s external validity, and they need to be considered (Wong, 2018). In any case, the municipality in question has many similarities with most Brazilian municipalities.

The main contribution of this study is twofold. On the one hand, we were able to describe in detail the problems concerning the quality of prescription writing that can jeopardize the safety of medication in primary health care. The quality of prescription writing is rarely considered in its different aspects when assessing medication safety. On the other hand, the set of indicators we use in our study can be viewed as a standard managerial tool to monitor the quality of prescription writing, at least in the context of Brazilian municipalities, to identify the local priorities for interventions to improve.

### Study limitations

A limitation of this study was the access to prescriptions in the DUs rather than directly from the patient. This collection alternative may have underestimated the prevalence of noncompliance with the indicator ‘medicine included in the institutional list officially approved’, as well as concerning the prescribed pharmacological classes.

## Conclusions

The quality of the evaluated prescriptions was very variable, both by prescribers and pharmacies. The situation shows, therefore, that there are many opportunities to improve the wording of the prescription.

This requires investigation, mainly about the most problematic indicators concerning noncompliance, so that more effective strategies are adopted to improve the quality of these prescriptions. That said, the trend is that this indicator also fits as a priority for planning strategies to improve the quality of drug prescriptions in primary care.

Low-quality prescriptions can compromise patient safety and the efficacy of drug therapy, and this impact needs to be evaluated in further studies.
